# Biotic and abiotic factors causing the collapse of *Robinia pseudoacacia* L. veteran trees in urban environments

**DOI:** 10.1371/journal.pone.0245398

**Published:** 2021-01-20

**Authors:** Agnieszka Wilkaniec, Beata Borowiak-Sobkowiak, Lidia Irzykowska, Włodzimierz Breś, Dariusz Świerk, Łukasz Pardela, Roma Durak, Jadwiga Środulska-Wielgus, Krzysztof Wielgus

**Affiliations:** 1 Department of Landscape Architecture, Poznań University of Life Sciences, Poznań, Poland; 2 Department of Entomology and Environmental Protection, Poznań University of Life Sciences, Poznań, Poland; 3 Department of Phytopathology, Seed Science and Technology, Poznań University of Life Sciences, Poznań, Poland; 4 Department of Plant Nutrition, Poznań University of Life Sciences, Poznań, Poland; 5 Institute of Landscape Architecture, Wrocław University of Environmental and Life Sciences, Wrocław, Poland; 6 Department of Experimental Biology and Chemistry, University of Rzeszow, Rzeszów, Poland; 7 Faculty of Architecture, Institute of Landscape Architecture, Cracow University of Technology, Kraków, Poland; USDA Forest Service, UNITED STATES

## Abstract

*Robinia pseudoacacia* L. is an interesting example of how one plant species can be considered invasive or useful depending on its environment. In the past this tree species was planted for decorative purposes and for wood in Poland. For many years it was recommended in poor and degraded habitats because it facilitated late-successional plant species. The aim of this study was to verify if black locust can still be regarded as a resistant tree species in urban greenery. The health condition of old tree specimens growing along streets and in parks was compared. The occurrence of pests and pathogens on *R*. *pseudoacacia* trees was assessed and the content of mineral elements in leaves was examined. The research results showed that the health of black locust trees growing in the urban environment in Polish cities, especially along streets (in comparison to park sites), deteriorated significantly due to the interaction of harmful biotic and abiotic factors. Increased level of toxic metals (Fe, Zn, Pb, Mn and Cd) in plant tissues and the accumulation of pests and pathogens negatively influenced the health of *R*. *pseudoacacia*.

## Introduction

The basic functions of greenery and the advantages of trees in urban ecosystems are widely known. They have positive influence on the city landscape and health-promoting effects for the city’s population. Usually the city is seen as one big ecosystem or as a complex of several individual ecosystems, e.g. parks and alleys [1]. The ecosystem functions of trees include decreasing ambient temperature, shading paved grounds, retaining rainwater, and removing air pollution [2–8]. It is broadly recognised that arborescent vegetation (tall greenery) can attenuate the urban heat island effect and build the resilience of cities to climate change [9]. The influence of trees on the range of ecosystem services is closely related to their physiological condition and capacity to persist within urban greenery [10]. Particularly large, old trees are important components of urban ecosystems [11,12]. They are known for influencing rich biodiversity [13] by providing microhabitats in their structure. Habitat structures provided by large, old trees take a long time to form and are not provided by younger trees. Relative to their size, they are disproportionate providers of resources crucial to other species [14]. Veteran trees also significantly contribute to ecosystem services (e.g., water balance, carbon sequestration, increasing air quality) [12,14–17]. Data collected by Hand et al. [18] in Great Britain demonstrate that the amount of carbon stored is low in young and semi-mature trees and highest in over-mature and veteran trees. Gross carbon sequestration rate also increases with tree age. The same research indicated that larger trees (usually older) with larger total leaf areas allow greater quantities of rainfall to be intercepted and that most examined species of trees showed an increased capacity for pollutant removal with increasing age. Old trees, which can be found in historical gardens, parks and alleys, are also important elements of cultural heritage.

Difficult urban conditions such as low humidity and extreme temperatures, dry seasons, bad soil structure, pollution, soil salinity, limited space for the development of tree roots and crowns, insolation, and strong winds in street canyons negatively affect the health of trees [7,10,19–21]. The urban environment significantly impedes the introduction of trees in cities but paradoxically, trees are necessary to improve the urban ecosystem. To avoid failures, tree species need to be carefully selected before they are planted in urban green spaces [19]. On the one hand, the importance of common species and ones that spontaneously appear in urban ecosystems is emphasised [22–24]. This is due to good adaptation to the local environment and positive ecological impact of native species. On the other hand, introduced species often have traits that facilitate their adaptation to adverse environmental conditions [7].

*Robinia pseudoacacia* L. (known as ‘black locust’ or ‘false acacia’) is an interesting example of how one plant species can be considered invasive in one environment and very useful in another. It is a medium-size (12-18m in height), hardwood forest tree developing extensive root systems. The radial root extent is about 1–1.5 times the tree height. The species originates from North America. It has spread worldwide due to its fast growth, rapid maturity [25], vigorous sprouting [26] and reproduction by underground runners [27]. Successful spreading of black locust is influenced also by the fact that disturbance promotes the growth of clones and leads to an increase in the number of ramets [27]. Black locust produces allelopathic substances in bark and roots that inhibit growth of some other plants [27]. *Robinia* trees have been widely planted, frequently escaped cultivation and therefore they are considered an invasive species in natural habitats [28]. Being an invasive, non-native tree, black locust is not planted in Polish forests anymore [29]. However, for a long time it was recommended in urban areas as its invasiveness was less important there [30–32]. Currently the planting of non-native, invasive species is not recommended worldwide not only in forests but also in urban green space [33]. Black locust is still a common tree in a large number of European cities [34,35]. Some specimens are old trees of high historical, aesthetical and landscape value. Sádlo et al. [36] proposed a stratified approach to *R*. *pseudoacacia* management, which accounts for both the ecological and economic aspects of its occurrence. An approach to *Robinia* in which the species is regarded as a noxious invader needs to be balanced with its integration into landscapes and wide social acceptance. Sjöman et al. [37] argued that as the availability of native tree species that are suitable for harsh urban environments depends on the richness of the regional tree flora. Regional context must be taken into consideration when selecting species. The limited abundance of Central European tree flora is discussed in the paper in the context of publications concerning Berlin, which is located 100 km away from the Polish border [37,38]. The main message from the line of arguments given by Sjöman et al. [37] is that we cannot afford to exclude non-native tree species from urban green space.

*R*. *pseudoacacia* has been present in Poland for more than 200 years [29]. It is a pioneer species, colonising poor and degraded habitats, even those where the soil cover has been disturbed and degraded [25]. For a long time, the tree has been recommended for urban areas in Poland because it facilitates late-successional plant species, especially because it is able to fix nitrogen [39] and stabilise the soil. Black locust is also useful in urban greenery due to its low sensitivity to most abiotic stressors [30–32,40]. Furthermore, it is believed that due to expected climate changes black locust may become prevalent in urban greenery as a species that is tolerant to extreme temperatures [41].

Black locust is usually considered to exhibit low susceptibility to diseases and pests because as an introduced species it probably has very few natural predators in Europe [27,42–45]. The “enemy release hypothesis” suggests that one of the reasons for rapid and successful invasions by non-native species might be their escape from natural enemies [46–49]. The lower herbivore pressure in Europe compared to the native North American range is thought to be a contributing factor to the effective spread of the black locust in the invaded area [26,27], in addition to its capacity for rapid reproduction. However, a significant number of *R*. *pseudoacacia* trees in Poland show symptoms of pest and fungal pathogen activity. Because some pathogens and pests develop well when temperature is low and humidity is high, while for others high temperature and low humidity are prerequisites, it seems obvious that climate warming may cause unexpected changes in their populations. Consequently, the health of their host plants will be affected [50,51].

The suitability of black locust for urban areas in Poland was described many years ago, but there have been no broader reports on new emergence of pests and diseases in a changing climate. The aim of this study was to verify if black locust could still be regarded as a resistant tree species in urban greenery in Poland. Which factors deteriorate the health of black locust most? Which biotic and abiotic factors interact together and increase the trees’ stress? In order to achieve the research objective, we evaluated the most important factors influencing the condition of black locust trees in selected urban areas of three Polish cities. The condition of trees growing along streets and in parks was compared. The occurrence of pathogens and pests on *R*. *pseudoacacia* trees was assessed and the content of mineral elements in leaves was examined.

## Materials and methods

### Study area

Poland is situated in Central Europe and has a typical temperate climate. Three locations in Poland (Kraków, Poznań and Wrocław) were chosen for the study. Most of the research sites were associated with 19^th^ century fortifications. Black locust trees were commonly planted there at the time when they were established [52]. Black locust was a very important species for fortification greenery throughout Central and Eastern Europe in the 19^th^ century.

The two research seasons differed in terms of climatic conditions. Year 2017 was more humid and cooler than 2018. Approximately a third more precipitation was recorded in the first research season than in the following one. June, July and August 2018 were particularly warm months. Kraków was the warmest city with the highest annual precipitation (Table 1).

**Table 1:** Monthly precipitation and average monthly temperature in the examined cities in 2017–18.

Year	Month	Monthly precipitation (mm)			Average monthly temperature (°C)		
2017		Poznań	Wrocław	Kraków	Poznań	Wrocław	Kraków
	Jan	7.6	13.5	8.2	-2.1	-3.2	-3.7
	Feb	30.2	25.7	34.8	0.8	1.2	2.1
	Mar	16.5	34.0	47.2	6.9	6.9	7.7
	Apr	28.1	63.8	102.0	7.9	8.0	8.9
	May	27.0	40.2	80.6	14.1	14.6	22.2
	Jun	48.4	65.2	61.8	18.1	19.1	22.2
	Jul	109.9	142.6	64.8	18.6	19.6	20.9
	Aug	92.9	64.1	80.6	19.3	19.9	21.6
	Sep	40.0	66.1	174.4	13.9	13.5	14.6
	Oct	67.1	71.4	89.2	11.4	11.1	10.9
	Nov	43.7	30.8	50.2	5.6	5.7	5.5
	Dec	45.1	27.1	32.8	2.9	3.0	3.1
2018	Jan	45.4	20.6	18.4	2.3	2.9	2.2
	Feb	5.4	2.8	9.0	-2.4	-2.4	-2.0
	Mar	27.4	26.5	21.0	1.0	1.2	2.1
	Apr	30.3	24.6	11.2	13.5	13.4	15.9
	May	15.2	49.4	40.2	17.4	17.1	18.6
	Jun	23.6	51.1	90.4	19.8	19.5	20.0
	Jul	82.1	72.9	156.4	20.6	20.8	21.3
	Aug	8.4	11.4	71.8	21.8	21.7	22.1
	Sep	37.2	51.2	77.0	16.6	16.1	17.3
	Oct	22.8	46.1	56.0	11.2	10.4	11.8
	Nov	5.4	12.5	9.6	7.8	5.3	5.4
	Dec	58.6	41.7	49.0	2.6	2.6	11.8

Source: Elaboration of authors based on data from the Wroclaw–Swojec Faculty of Agrometeorology and Hydrometeorology Observatory (WOAiHW-S), Davis VantagePro2 weather station in Kraków and the meteorological station at the Poznań - Ławica airport.

Since the 1970s, a gradual increase in temperature has been observed in the cities covered by the research. The average annual air temperature in the studied years in relation to the average of the decade 1971–1980 was approximately 3°C higher in Poznań and Wrocław and 4°C higher in Kraków (Table 2). Changes in the annual values of observed mean precipitation in Poland in the last 5 decades were statistically insignificant, at the 10% level. Number of days with intense precipitation was found to increase especially in the northwestern part of Poland, while the increase in the number of consecutive dry days was noted in the summer half-year [53].

**Table 2:** Mean annual values of air temperature (^o^C) in 2017–18 and in decades of period 1971–2000 in individual locations.

City	1971–80	1981–90	1991–00	2017	2018
Poznań	8.2	8.5	8.8	9.78	11.02
Wrocław	8.3	8.7	9.1	9.95	12.07
Kraków	7.6	8.1	8.5	11.33	12.21

Source: Elaboration of authors based on data from 1971–2000 described by Michalska [54], data from 2017, 2018 provided by the Wroclaw–Swojec Faculty of Agrometeorology and Hydrometeorology Observatory (WOAiHW-S), Davis VantagePro2 weather station in Kraków and the meteorological station at the Poznań - Ławica airport.

Two locations were selected in each city: a ‘park’ site–with trees growing in a place located away from busy roads, other sources of pollution and densely built-up areas, with no paved surfaces or vehicles traffic under the crowns of trees, and a ‘street’ site–next to main roads with heavy traffic, where salt is used for road maintenance in winter, partly paved surfaces and parking sites or roadway within the range of tree crowns.

The park sites included the slopes of Rajsko Fort in Krakow (49°59'20.9"N 19°58'08.7"E), Fort VIIIa (Jasińskiego Park; 52°23’09.17”N, 16°52’01.39”E) in Poznań, and Infantry Shelter No. 20 near Wiaduktowa Street in Wrocław (51°4’26”N, 17°4’22.8”E). The street sites were the slope of Pszorna Fort crossed by Jana Pawła II Avenue in Krakow (50°04’21.45”N, 19°59’22.85”E), part of the former fortress road, Lutycka Street (road between Szczawnicka and Podolańska Streets; 52°26’17.53”N, 16°53’24.34”E) in Poznań and alley plantings from the 1930s in Wyszyńskiego Street near Tołpy Park in Wrocław (51° 7' 12.5"N, 17° 3' 6"E). The street site in Wrocław was the only site not located in historical 19^th^ century fortifications.

The research was conducted on 10 specimens of black locust (*Robinia pseudoacacia* L.) in each site (60 specimens in total). The age of the trees was estimated at 70–130 years, by assessing the diameter of the trunk [55,56] and information on the date of the construction of the fortification objects they accompanied. The median of their circumferences was 206.6 cm. The trees selected for the study were several decades old so that the assessment of their health would not be impeded by the problems of young trees adapting to a new site and so as to focus the analysis on the most valuable specimens in the urban environment.

A permit was obtained from the Municipal Green Space Department to conduct research in Poznań. Obtaining a permit in other cities was not required.

### Evaluation of condition of trees

The overall condition of the trees was evaluated with the visual method invented by Kosmala et al. [42], which is a modification of the Roloff method [57]. If experienced people visually evaluate the condition of trees, the method gives objective and repeatable results [42,58]. The visual method enables evaluation of the condition of the crown (branches, shoots and leaves), trunk, and roots of the tree. The parts of the tree are evaluated on the basis of two features: defects in tree structure and symptoms of diseases. The structure of the crown is assessed based on the degree of defoliation, its vitality as well as pest infestation and infection with pathogens. The assessment of the trunk concerns its structure (bark damage, wood defects and cracks) and disease symptoms. The method also takes into account the assessment of the state of the tree's root system by the state of buttresses and structural roots, as well as the state of proper roots based on soil observation. Individual features of the tree related to its state have been transformed into points. The results obtained are used to calculate the total condition of the tree expressed as a point value which is a sum of the crown, trunk and root zone condition assessment. The degree of damage is described as a percentage share of the total condition. The tree can have a maximum score of 100 points, which means 100% of health (30 points for the condition of the crown, 30 points for the condition of trunk and 40 points for the condition of the roots), on a five-point scale, where 0–15 points means very poor condition, 16–45 poor condition, 46–75 average condition, 76–95 good condition, 96–100 very good condition. The final stage of assessment is to determine the vitality phase of the trees (very good and good—expansion phase, medium degree—weakness, bad degree—stagnation, very bad degree—resignation phase). The condition of the trees in all three cities was evaluated in August 2018.

### Chemical analysis of mineral components in leaves

In late July 2018, 20 leaves were collected from each tree in each site: random samples from 2–5 branches on different sides and at different heights (1–3 m) in the crown, with single leaves collected from different places on the branches (top and middle). The leaves were analysed chemically to assess the influence of the environment on the quality of the plants. First, dust and other impurities were mechanically removed from the leaf surface. Next, the samples were pre-dried at a temperature of 50°C and ground in a mixer. In order to assay the total content of N, P, K, Ca, Mg, and Na the plant material was digested in concentrated sulphuric acid with hydrogen peroxide. To analyse the total Fe, Mn, Zn, Cu, Cd, and Pb content the plant material was digested in a mixture of concentrated (ultrapure) nitric and perchloric acids (analytically pure) at a 3:1 ratio. After digestion the following assays were made: the total N content was assayed with the micro-Kjeldahl procedure; the P content was analysed with spectrophotometric methods; the concentrations of K, Ca, and Na were measured by means of flame photometry; and Mg, Fe, Mn, Zn, Cu, Pb, and Cd content were measured by means of flame atomic absorption spectrophotometry [59]. The accuracy of the measurements of heavy metals was tested by analysis of reference material certified by the Institute for Reference Materials and Measurements (IRMM), Belgium. The chemical composition of the leaves was analysed separately for each tree.

### Leaf sampling and evaluation of biotic factors affecting health of trees

In order to evaluate the threat of infestation of the *R*. *pseudoacacia* trees with harmful fauna and pathogenic fungi, ten specimens were selected in the park and street sites in each city. Between 2017 and 2018, from early May to late September samples of 10 compound leaves were collected from each of 60 trees (in total 600 leaves were assessed twice a month). Leaves from each tree in each site (10 compound leaves per tree) were collected randomly: samples from 2–5 branches on different sides and at different heights (1–3 m) in the crown; single leaves collected from different places on the branches (top and middle).The samples were taken to a laboratory, where the leaves were analysed. Fungal pathogens were identified on each leaf based on disease symptoms and typical morphological traits. The cause of damage was identified based on developmental stages or symptoms of pest feeding. Damage caused by biting species of insects (mines and bitten holes) were counted on each leaf. For piercing-sucking species of insects, specimens were counted. Next, the number of specimens of a given insect species and the damage they caused and disease symptoms caused by fungal pathogens were summed up for each tree (sum from 10 leaves).

### Statistical analysis

Statistical analyses were preceded by the Shapiro-Wilk test to check the data distribution. The data used in the study are characterized by a normal or close to normal distribution.

The influence of location (park, street) on chemical compositions of leaves was evaluated using analyses of variance (ANOVA). To assess the differences between means, the Tukey test (p ≤ 0.05) was used. Standard deviation was also calculated.

The statistical analyses and models were created using discriminant analysis. The analyses checked which variables (macro- and microelements identified in the leaves of the trees growing at different sites) may affect the health of trees (Q) and the occurrence of diseases and pests. The analyses also determined which species of pests and pathogens were more numerous at the research sites. Canonical variate analysis (CVA), a canonical form of Fisher's linear discriminant analysis (LDA), was used to construct a model [60].

**Fig 1:**
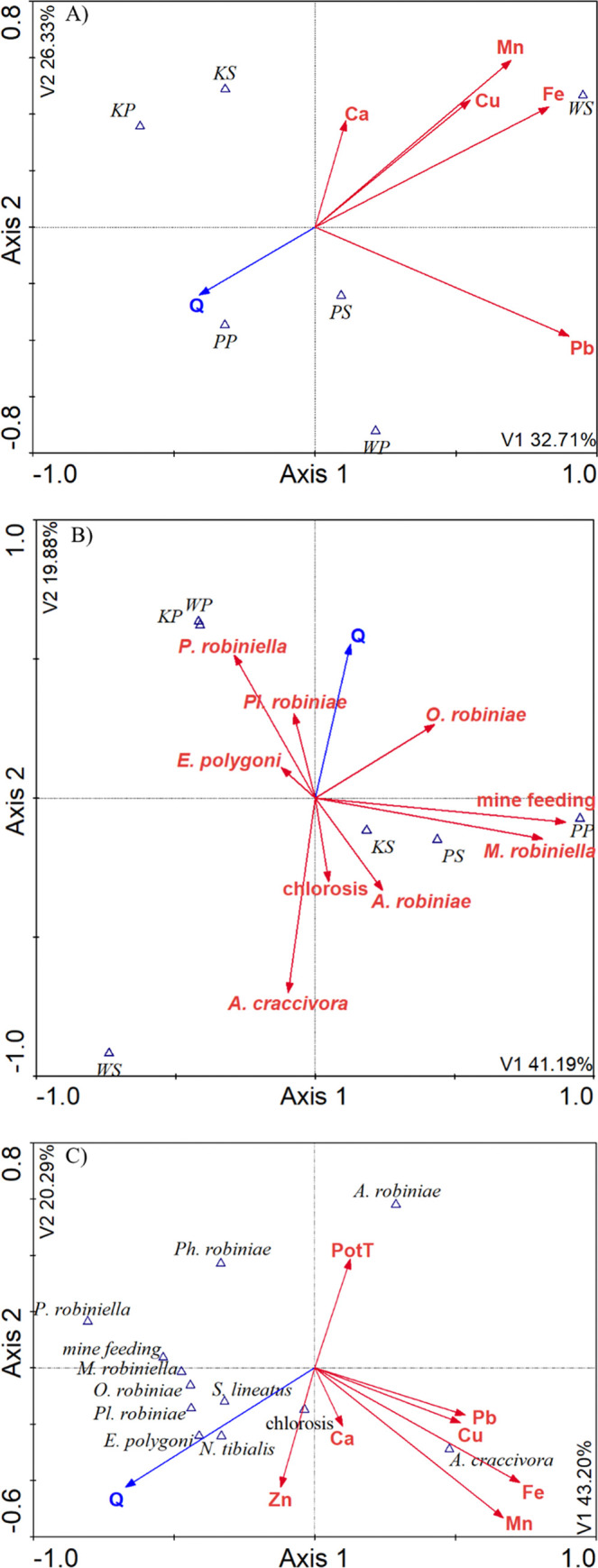
CCA analysis (n = 60). The distribution of micro- and macroelements in the black locust leaves according to location. A). The distribution of black locust pests and pathogens in the locations under study and their effect on the health of the trees—B). The influence of micro- and macroelements in black locust leaves on the distribution of diseases and pests in relation to the health of the trees—C). KP—Krakow park; PP—Poznan park; WP—Wrocław park; KS—Krakow street; PS—Poznań street; WS—Wrocław street; *Aphis craccivora–A*. *craccivora; Appendiseta robiniae–A*. *robiniae; Macrosaccus robiniella–M*. *robiniella; Parectopa robiniella–P*. *robiniella; Obolodiplosis robiniae–O*. *robiniae; Nematus tibialis–N*. *tibialis; Sitona lineatus–S*. *lineatus; Erysiphe polygoni–E*. *polygoni; Pleospora robiniae–Pl*. *robiniae; Phylosticta robiniae–Ph*. *robiniae;* chlorosis; Q-health quality; PotT–perimeter of tree; n–number of trees.

The CVA resulted in 3 canonical correspondence analysis (CCA) models (Fig 1). Stepwise regression analysis was used to find which variables had the greatest influence on the health of the trees at the research sites. All variables were assessed. The ones that contributed most to the discrimination of the groups based on the p and F values for the variable under analysis were included in the model. The process was repeated until the p value fell below 0.05 for the variable under analysis.

The Monte Carlo permutation test was applied (number of permutations 999) in order to determine the significance level. Canoco for Windows and Microsoft Excel spreadsheets were used for all comparisons, calculations and graphic elements. The following tools from the Canoco for Windows package were used: Canoco for Windows 4.5, CanoDraw for Windows and WCanoIMP.

## Results

### Evaluation of tree condition

The health condition of the trees growing at the park sites was generally better than those growing at the street sites (Table 3), which was statistically confirmed for Poznań and Wrocław. The average score of the park sites was 85.7 points (85.5 in Krakow, 85.4 in Poznań, 86.2 in Wrocław; good condition, expansion phase), whereas at the street sites it was 75.5 points (81.3 in Krakow, 74 in Poznań, 71.3 in Wrocław, average condition, weakness phase).

**Table 3:** The health of black locust trees.

	Park site				Street site				T-test
	Min	Max	X	SD	Min	Max	X	SD	<0.05
Poznań	69	100	85.4 a	9.70	31	89	74 b	17.11	0.0439
Kraków	77	93	85.5 a	4.25	70	87	81.3 a	6.46	0.0529
Wrocław	70	92	86.2 a	6.96	58	83	71.3 b	8.43	0.0002

Min—minimum, Max—maximum, X (n = 10)–mean, SD—standard deviation.

### Chemical composition of leaves

The foliage of the trees growing in the areas adjacent to streets and in parks did not differ in content of nitrogen, phosphorus (except Wrocław), potassium, magnesium, or copper (Table 4A–4C). In all the cities the leaves of the plants exposed to higher levels of pollution caused by heavier car traffic contained significantly greater amounts of calcium, sodium, iron, zinc, and lead. There were similar dependencies observed for manganese and cadmium. Only in Wrocław the differences in the content of these elements in the leaves of the trees growing at the street and park sites were so big that they were statistically significant. The largest site-dependent differences in the chemical composition of leaves were found in Wrocław. For example, in comparison with the trees growing in the park, the samples of plant material collected from the trees growing along the streets contained 96% more iron and 444% more manganese. In Kraków and Poznań the differences were much smaller and did not exceed 60% for iron and 24% for manganese.

**Table 4:** A. The chemical composition of *R*. *pseudoacacia* leaves grown in Kraków. B. The chemical composition of *R*. *pseudoacacia* leaves grown in Poznań. C. The chemical composition of *R*. *pseudoacacia* leaves grown in Wrocław.

		Park				Street			Anova
			% dry weight						p-value
	Min	Max	X	SD	Min	Max	X	SD	<0.05
N	3.08	4.34	3.72 a	0.38	3.33	4.34	3.81 a	0.27	0.358
P	0.14	0.22	0.20 a	0.02	0.16	0.25	0.20 a	0.02	0.896
K	1.09	2.63	1.60 a	0.42	0.35	2.31	1.52 a	0.49	0.522
Ca	1.05	1.86	1.41 a	0.25	0.92	2.56	1.71 b	0.42	0.002
Mg	0.12	0.20	0.17 a	0.02	0.14	0.32	0.24 a	0.06	0.078
			mg kg^-1^ dry weight						
Na	20.0	50.0	40.0 a	10.00	30.0	90.0	60.0 b	20.00	0.021
Fe	60.4	109.3	79.3 a	13.24	92.1	173.5	123.2 b	24.83	0.001
Mn	16.1	68.1	38.2 a	13.99	20.4	78.7	47.1 a	22.84	0.087
Cu	6.2	18.3	9.1 a	3.29	6.9	11.9	8.9 a	1.47	0.987
Zn	18.8	40.0	27.5 a	6.89	18.8	76.3	44.6 b	15.08	0.002
Pb	2.8	5.3	4.1 a	0.89	4.1	10.6	6.5 b	1.68	0.034
Cd	0.04	0.32	0.20 a	0.08	0.05	0.52	0.28 a	0.15	0.065
N	3.08	4.20	3.69 a	0.32	3.15	4.69	3.76 a	0.42	0.098
P	0.15	0.23	0.19 a	0.03	0.15	0.24	0.21 a	0.03	0.874
K	1.06	2.00	1.65 a	0.32	1.26	2.25	1.62 a	0.27	0.426
Ca	0.63	1.70	1.14 a	0.33	0.96	2.67	1.57 b	0.51	0.037
Mg	0.13	0.20	0.17 a	0.02	0.14	0.20	0.17 a	0.02	0.986
	mg kg^-1^ dry weight								
Na	50.0	70.0	60.0 a	10.0	60.0	110.0	80.0 b	20.00	0.012
Fe	67.1	97.7	77.5 a	8.58	105.0	137.7	124.1 b	8.41	0.001
Mn	22.1	41.0	29.1 a	4.82	17.2	44.6	34.9 a	15.79	0.089
Cu	4.6	11.1	7.3 a	1.91	4.7	8.6	6.5 a	1.18	0.126
Zn	15.1	25.3	19.8 a	3.21	21.4	42.3	31.4 b	7.14	0.001
Pb	6.7	11.8	8.9 a	1.77	9.4	15.9	12.4 b	2.14	0.042
Cd	0.08	0.50	0.32 a	0.14	0.25	0.46	0.38 a	0.07	0.251
N	3.22	3.78	3.53 a	0.19	3.08	3.64	3.42 a	0.15	0.896
P	0.17	0.22	0.19 a	0.01	0.19	0.27	0.23 b	0.03	0.041
K	1.69	2.72	2.08 a	0.32	1.59	2.55	1.95 a	0.26	0.062
Ca	0.59	1.61	1.07 a	0.31	0.58	2.71	1.67 b	0.57	0.015
Mg	0.19	0.27	0.23 a	0.04	0.18	0.31	0.24 a	0.03	0.896
	mg kg^-1^ dry weight								
Na	60,0	110.0	90.0 a	20.00	70.0	170.0	120.00 b	30.00	0.001
Fe	92.9	129.7	107.9 a	11.74	147.2	307.7	211.6 b	49.05	0.003
Mn	17.5	35.6	25.1 a	5.17	55.1	207.2	135.5 b	44.78	0.001
Cu	6.1	10.5	8.2 a	1.32	6.7	10.5	9.1 a	1.14	0.078
Zn	23.7	54.3	35.3 a	10.04	33.8	89.3	55.9 b	18.43	0.002
Pb	11.1	17.1	13.9 a	1.73	11.9	16.8	15.5 b	1.54	0.035
Cd	0.12	0.40	0.31 a	0.08	0.04	0.74	0.44 b	0.19	0.011

Min—minimum, Max—maximum, X (n = 10)–mean, SD—standard deviation.

Results of the CCA showed dependencies between: (A) the content of elements in leaves (Ca, Mn, Cu, Fe, Pb), the location of the trees in parks or streets, and their health (Q); (B) pests and diseases occurring on the trees, the location of the trees in parks or streets, and their health (Q); (C) the content of elements in leaves (Mn, Fe, Cu, Pb, Ca, Zn), the perimeter of the trees, pests and diseases occurring on the trees and their health (Q) (Fig 1). The strongest dependencies between the factors were found in Wrocław; the content of Fe, Mn and Cu in the leaves had the most negative effect on the health of the black locust trees growing along the streets of this city (Fig 1A). The remaining dependencies are discussed in the next parts of the paper.

### Biotic factors affecting health of trees

**Fig 2:**
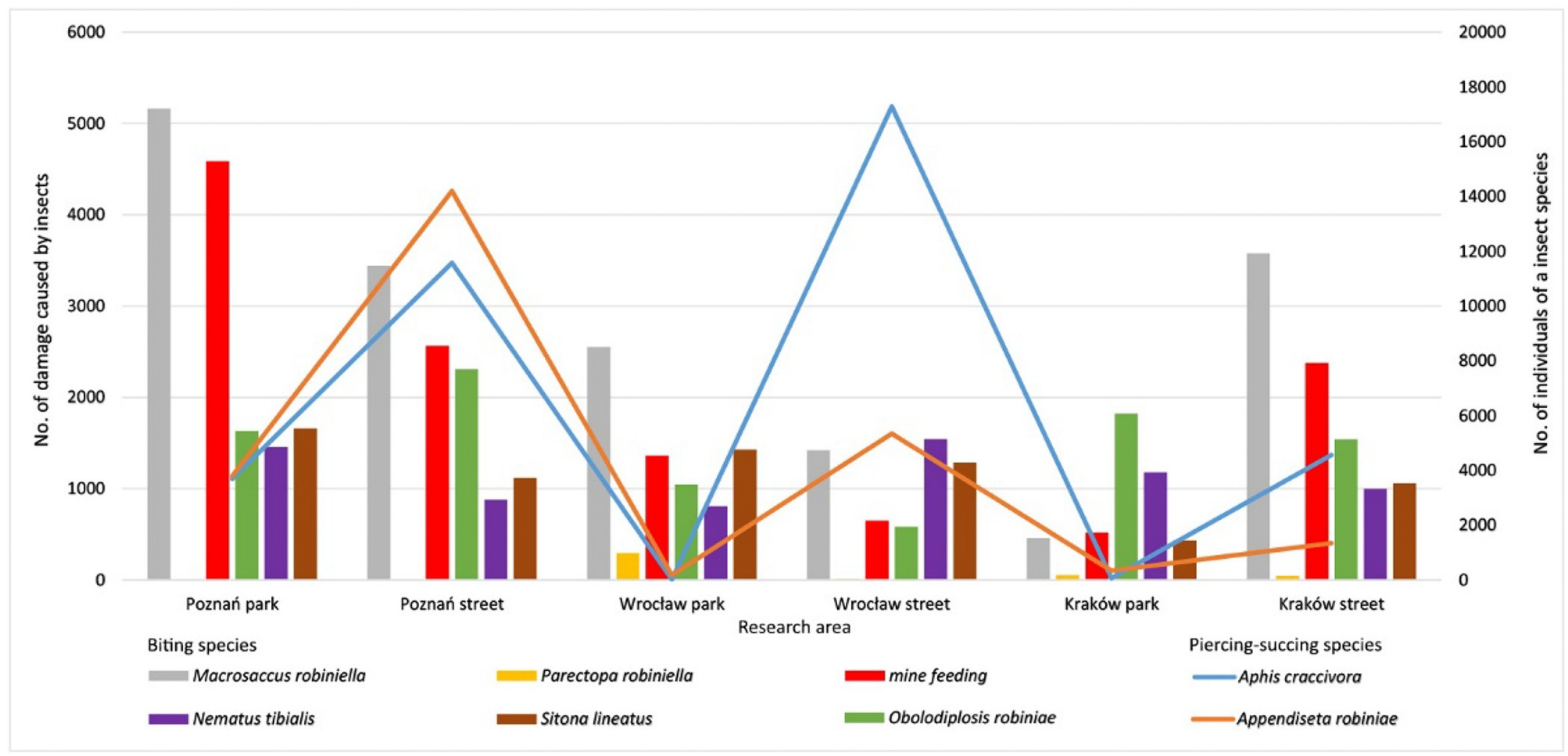
Damage to black locust trees caused by biting insects and the number of piercing and sucking insects found on leaves in 2017–2018.

The composition of the entomofauna was the same in all of the research sites, but they differed in the intensity of pest infestation. There were symptoms of damage to black locust leaves caused by insects with biting mouthparts and by those with piercing and sucking mouthparts. Representatives of 5 orders of insects were identified (Fig 2). There were two species of aphids of the Hemiptera order: *Aphis craccivora* Koch. and *Appendiseta robiniae* (Gilette). There were two leaf-mining insects of the Lepidoptera order: *Macrosaccus robiniella* (Clemens) and *Parectopa robiniella* (Clemens). *Obolodiplosis robiniae* (Haldeman) of the Diptera order, *Nematus* (Pteronidea) *tibialis* Newman of the Hymenoptera order and *Sitona lineatus* L. of the Coleoptera order bit the leaf tissue. During the observation, in addition to leaves, we examined trunks and low-lying tree branches, but no insect damage to these parts of trees was found.

The populations of aphids at the street sites in all the cities were more numerous than at the park sites. However, insects with biting mouthparts caused more damage to the leaves of the trees growing at the park sites. Apart from aphids, *M*. *robiniella* was the dominant species colonising *R*. *pseudoacacia* trees. There were larger numbers of these butterflies found on the leaves of the trees growing in the parks. Only in Krakow was more damage found at the street site. *O*. *robiniae* was also identified as a significant pest of black locust trees. The larvae of these flies fed on the leaves and caused the lamina edges to curl. As a result, the tissue began to crumble and dried out earlier. This species was more common at the park sites. Only in Poznań the trees growing in the street site were slightly more damaged.

The insects feeding on *R*. *pseudoacacia* significantly affected the condition of the trees, mainly those growing along the streets. The CCA showed that both aphid species had the greatest correlation with the Q parameter (Fig 1B and 1C).

**Fig 3:**
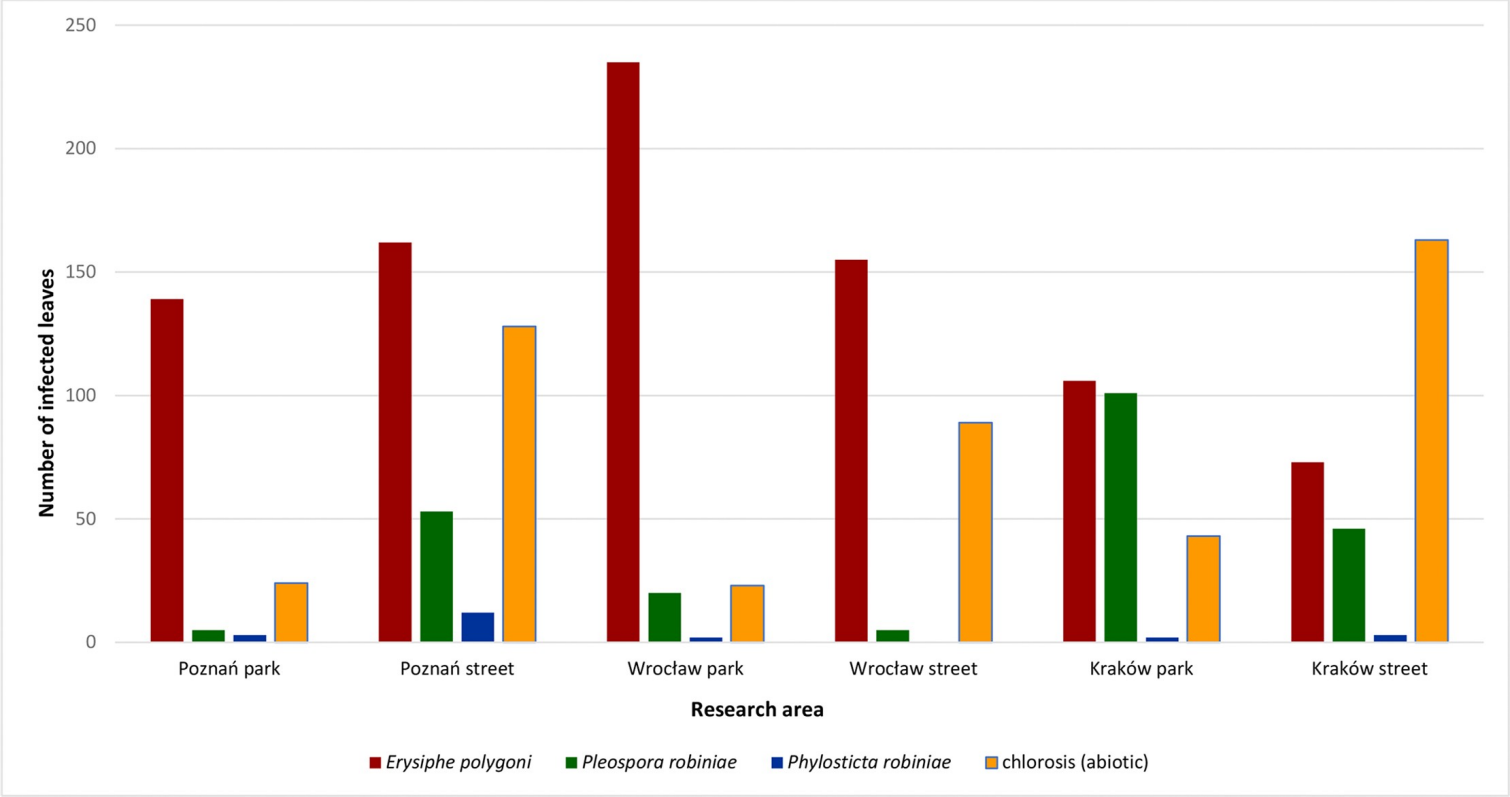
The occurrence of fungal infections and chlorosis of black locust trees in 2017–2018.

There were symptoms of fungal diseases observed on *R*. *pseudoacaccia* leaves in all of the sites. They significantly reduced the decorative value of the trees and deteriorated their health (Fig 1B and 1C) (Table 3). The leaves of the trees growing both in the parks and along the streets were infected by pathogenic fungi of the following species: *Pleospora robiniae* Lib., *Phyllosticta robiniae* Sacc. and *Erysiphe polygoni* D.C. (Fig 3). The *Pl*. *robiniae* fungus caused the necrosis and deformation of black locust leaves from early spring onward. The trees infected by *Ph*. *robiniae* had black spots on their leaves. However, this pathogen had weaker correlation with the Q parameter than *Pl*. *robiniae*. *E*. *polygoni* was the dominant pathogenic fungus in all locations (Table 5). It caused powdery mildew, which was manifested by the occurrence of white mycelium on the surface of leaf blades. The pathogen limited access to light and thus, it negatively affected the dynamics of photosynthesis and the condition of the trees (Fig 1). *E*. *polygoni* infections were more commonly found on the leaves of the trees growing at the park sites. Only in Poznań the fungus was more frequent at the street site.

**Table 5:** Statistical parameters for CCA models.

Model A				Model B				Model C			
number of included variables: 13				number of included variables: 13				number of included variables: 11			
number of rejected variables: 1				number of rejected variables: 0				number of rejected variables: 3			
number of permutations: 999				number of permutations: 999				number of permutations: 999			
	E %	P	F		E %	p	F				
Fe	12.63	0.001	8.619	*A*. *craccivora*	13.02	0.001	6.735	Q*	15.52	0.001	13.256
Pb	10.11	0.001	6.381	*M*. *robiniella*	11.33	0.001	5.857	Fe	11.18	0.001	10.569
Zn	9.04	0.001	4.132	mine feeding	9.77	0.001	4.949	Mn	10.25	0.001	9.201
Na	8.74	0.001	3.502	Q*	8.41	0.002	5.004	Mg	8.95	0.001	8.556
Q*	8.23	0.001	3.498	*P*. *robiniella*	7.51	0.002	3.621	Zn	8.12	0.001	7.236
Mn	6.11	0.006	2.594	*Pl*. *robiniae*	6.12	0.001	3.588	Pb	7.74	0.002	6.632
N	6.03	0.019	2.187	*O*. *robiniae*	6.07	0.001	3.326	Cu	7.55	0.009	6.445
Ca	5.91	0.029	2.132	*A*. *robiniae*	5.69	0.002	3.251	PotT**	6.69	0.014	5.996
PotT**	5.79	0.031	2.089	*Ph*. *robiniae*	5.22	0.009	3.089	Cd	6.23	0.020	3.121
Mg	5.55	0.034	2.085	PotT**	5.09	0.015	2.956	Na	5.89	0.030	2.441
Cd	5.23	0.040	2.079	*S*. *lineatus*	5.04	0.019	2.796	Ca	5.40	0.039	2.238
K	4.77	0.045	2.039	*N*. *tibialis*	4.72	0.026	2.563				
Cu	3.98	0.049	1.998	*E*. *polygoni*	4.11	0.046	2.244				

*Q-health quality

**PotT–perimeter of tree.

Apart from that, chlorosis was identified on leaves in all the locations, but it was much more pronounced at the street sites (Fig 3). The effect may have been caused by pollution (Fig 1).

## Discussion

Black locust is an important species in historical parks, gardens, alleys and fortifications in Poland. Old *R*. *pseudoacacia* trees might also be desirable because they provide shade and habitat for rare saprophytic fungi and saprophagous beetles [36,61,62]. *R*. *pseudoacacia* trees growing in Europe prefer high insolation, but there are no specific requirements concerning soil pH or fertility [63]. It is widely believed that black locust trees can adapt to drought [64], air pollution [65] and strongly acidic soils [66]. For the above reasons, it seems to be an interesting species from the point of climate change and the functioning of trees in urban environments, and why we chose it as a model species. However, our research indicates that the health of this species in Poland was visibly worse in the street sites than in the park sites. It can be assumed that this was related to adverse abiotic and biotic factors occurring in street locations as well as indirectly to global warming. In Poland, a gradual increase in temperature has been observed since 1987. According to Kożuchowski and Żmudzka [67], warming of 0.9°C in 50 years has manifested in particular as an increase in temperature at the turn of winter and spring, especially February and March. Winter and summer have shortened, and the transitional seasons have extended, which also has contributed to the gradual lengthening of the growing season [54]. This has resulted in extension of the period of plant colonization by pests and pathogens and increases in the range of invasive species as well as the number of generations for insects [68–71]. In the last 20 years of the 20th century and in the present decade, significant warming has also been observed in the winter and spring seasons, as well as in the summer [72]. Further climate warming is predicted. According to Kundzewicz et al. [53], the mean annual temperature in Poland is expected to increase by about 1°C until 2021–2050 and by about 2°C until 2071–2100. The warming is projected to be stronger in winter than in other seasons.

As the climate is changing, there is a wider spectrum of insects and pathogenic fungi colonising black locust trees, which have been considered as resistant to diseases and pests according to the “enemy release hypothesis” for introduced species so far. Time and changing environmental conditions can break resistance to an invader [73,74]. After over 200 years presence in Poland and climate change, black locust is damaged by a broader spectrum of pests and diseases than before. Many pests new to Poland (4 species versus 7 noted) have appeared on *Robinia pseudoacacia* in the last 20 years, which coincides with climate warming. Most of the pest species found on black locust trees are alien to the fauna of Poland. They are native to North America. The deformations of black locust leaves and the negative effects on their decorative values were mainly caused by aphids, flies and butterfly larvae. *A*. *robiniae* and *O*. *robiniae* were first found in Poland in 2007 [75,76], whereas *M*. *robiniella* and *P*. *robiniella* were found in the last decade of the 20^th^ century [77]. Although these species appeared in Poland a relatively short time ago, they acclimatised and are expanding northwards. This phenomenon may have been caused by global warming [78]. In many European countries, including Poland, monophages from America are listed as the most important pests of *Robinia*: false acacia gall midge *O*. *robiniae*, mining butterflies *M*. *robiniella* and *P*. *robiniella*, and *A*. *robinie* aphid [26,79]. Therefore, the question arises whether we can expect other North American species associated with this plant to appear in Poland along with global warming. It seems that *Metcalfa priunosa* (Say) may migrate to Poland. This is a polyphagous insect that feeds not only on fruit plants but also on shrubs and woody plants, including *R*. *pseudoacacia*. It is an invasive alien species that is widely distributed in Europe. It was first found in Italy in 1979 and rapidly invaded other countries: France, Spain, Switzerland, Austria, Bulgaria, Greece, and Serbia [80]. So far this insect has not been found in Poland, but it has appeared in neighbouring countries such as Germany, the Czech Republic, Ukraine, and Slovakia [81]. Due to the rapid adaptation and expansion of this species, its population is expected to increase and it may invade a wider range of host plants [82]. Important *R*. *pseudoacacia* pests in North America are wood-damaging insects: *Megacyllene robiniae* (Forster) (belonging to the Cerambycidae family) and the butterfly of the Cossidae family—*Prionoxystus robiniae* (Peck). These species have not been recorded in Europe so far [83]. Black locust wood can be damaged also by beetles of the Cerambycidae family: *Plagionotus arcuatus* (L.), *Clytus arietis* (L.) or *Chlorophorus figuratus* (Scopoli). They are polyphages found in central and southern Europe [79], but no damage caused by these insects was noted at the examined sites.Due to global warming, species previously found in southern Europe are migrating north and appearing in Poland, where they often winter and reproduce. Also, native species that occurred locally, especially in warm places, are quickly spreading throughout the country. Migration is facilitated by human activities, especially those related to the transport of plants, and by the lack of natural predators. In recent years new pests and diseases have also emerged on other tree and shrub species introduced in Poland, such as the horse chestnut and boxwood. Horse chestnut trees (*Aesculus hippocastanum* L.) are invaded by the horse chestnut leaf miner (*Cameraria ohridella* Deschka & Dimić) and *Erysiphe flexuosa* fungus (Peck) U. Braun & S. Takamatsu, which causes powdery mildew and serious health problems. *Aesculus hippocastanum* is an important ornamental tree species, which has been planted in parks and gardens in Poland since the 17^th^ century. *C*. *ohridella* was first found in Poland in 1998, whereas *E*. *flexuosa* in 2002. Before these dates they were listed in other European countries [84]. The expansion of *C*. *ohridella* to new areas may have been caused not only by its importation but also by the shift of the northern and eastern limits of its range as a result of global warming [78]. As the climate change progresses, its range is forecast to increase further [85]. Currently, *Cydalima perspectalis* (Walker), which was brought to Europe with boxwood seedlings, is a big problem. The species is native to humid subtropical regions of Southeastern Asia. In Europe it was first found in Germany in 2006. Then it spread rapidly to become an invasive species found in most Western and Southern European countries [86]. In Poland it was first observed in 2012 [87]. In view of the adaptability of this butterfly and the global rise of temperatures, the range of this pest is forecast to extend to the northern borders of Europe [88]. Thus, the expansion *of C*. *perspectalis* is determined by temperature rather than humidity, because this pest has perfectly adapted to a much drier climate in Europe.

The results of research conducted in three Polish cities show that there were larger numbers of insects with piercing and sucking mouthparts found on the plants growing along the streets. By contrast, the black locust trees in the parks usually had more insects with biting mouthparts. This observation was in line with the findings of the research conducted on other common tree species in Poland, i.e. maple and lime-trees [89,90]. The authors of those studies found much larger populations of aphids on trees growing along streets than on the ones growing in parks. The pressure of urbanisation as well as particulate matter and harmful substances from exhaust gases cause the number of phytophages with biting mouthparts to decrease, while the number of piercing-sucking species is increasing [89,90].

The results of our research show that neither salinity nor higher concentration of metals (Fe, Mn, Cu and Pb) were correlated with development of aphids. There were similar findings of studies on the occurrence of aphids in many other urban environments [91,92]. These results do not correspond to the findings of research on *Tilia* 'Euchlora' conducted by Sienkiewicz-Paderewska et al. [93]. Those authors investigated the effect of salt stress on lime aphids and observed larger numbers of these insects on trees growing in parks. This suggests that lime aphids preferred the leaves of plants with low content of Cl and Na, but with higher concentration of N.

There were symptoms of fungal diseases on the black locust trees examined in our study. They were caused by the following pathogens: *Pl*. *robiniae*, *Ph*. *Robiniae*, and *E*. *polygoni*. Each of these species had a specific adverse effect on the decorative values of *R*. *pseudoacacia* trees. They caused unsightly discoloration of leaves, premature defoliation or a mycelial cover on the surface of leaves. There were similar observations made in research on the occurrence of black locust pathogens in Germany [94].

*E*. *polygoni* fungus, which causes powdery mildew, was usually dominant at all the research sites. Powdery mildew is initially manifested by small, white patches on leaves. Later fungal colonies expand and cover the leaf surface. Because there were two rather dry growing seasons during the research, the dominance of *E*. *polygoni* seems understandable. Most species of fungi thrive better at higher humidity because their spores need drop of water to germinate. However, the fungi causing powdery mildew on various plant species are an exception, as they develop very expansively when the growth of other fungi is inhibited by the shortage of water [84]. It is noteworthy that when black locust trees are infected by the polyphagic *E*. *polygoni* fungus, apart from their own symptoms, they may also be a source of inoculum, which can infect other species of plants in the urban environment. There have been few scientific reports on the health of black locust trees because this species is considered invasive and undesirable in many habitats [44,95].

Apart from the adverse effects of biotic factors, chlorosis of black locust leaves was also observed. It was particularly intensive at the street sites due to the high content of some metals in the leaves. The high content of Zn seems to be particularly closely correlated with the occurrence of chlorosis (Fig 1).

The research showed that the Fe, Zn, and Pb content in the leaves of the trees growing along the streets was significantly higher than in the samples collected from the trees growing in the parks. There was a similar observation made about the content of Mn and Cd. Apart from the Mg content, the content of other elements measured in Kraków, Poznań and Wrocław was comparable with the values measured by other authors [96–98].

The assessment of our research findings looks different when the results of the analyses are compared with the optimal or toxic content elaborated for deciduous trees by Jones et al. [99] and by Kabata-Pendias and Pendias [100]. The measurements made on the trees growing along the streets in Wrocław showed that the content of elements was toxic (Fe and Pb), elevated (Zn) or it exceeded the optimal amount (Mn). The content of Pb in the samples of plants growing in the park in Wrocław and in the street in Poznań was also toxic. However, the content of cadmium in the *R*. *pseudoacacia* leaves was elevated, but not toxic.

Due to heavy traffic street areas are usually more exposed to pution than parks. However, in Wrocław there was high content of metallic elements in the leaves of the plants growing at the park site. It may have been caused by the fact that the research site was located close to a freight bypass railway, which has been in operation since around 1891.

The results of chemical analyses of the leaves collected from the plants in Kraków, Poznań and Wrocław showed that despite the diverse pressure from human activities, the influence of the location of *R*. *pseudoacacia* trees on the accumulation of macroelements (except calcium) was mostly insignificant.

The leaves of the trees growing along the streets had increased content of calcium. This effect may be caused by–as often documented in literature–higher concentrations of Ca in the street soil samples than in the park soil samples [101,102]. In Poland fly ash, which is formed when coal is burnt in furnaces, is one of the causes of the high content of calcium in urban soils. It flies in the air in the form of alkaline particulate matter. This problem is intensified in the winter during the heating season. Depending on the quality of coal, fresh ashes contain 2.95–33.25% of CaO [103]. When fresh, these are strongly alkalising compounds. Even soils which have never been limed but are located in urban areas are rich in calcium [104].

In all three cities the content of Na in the leaves of the black locust trees growing along the streets was only slightly higher (20–30 mg·kg^-1^) than in the leaves of the park trees. The amounts of sodium measured in the dry matter of plants were divergent and ranged from 30 [105] to 460 mg·kg^-1^ d.w. [106]. In this context, the Na content in the leaves analysed in our study was very low. Because sodium chloride is used in winter, the higher content of Na in the leaves of trees growing along streets was often documented in scientific publications [107,108]. However, recently NaCl has been used very sparingly due to snowless winters for many years and improved ecological standards of road maintenance implemented by municipal governments. As a result, the sodium content in plants is constantly decreasing.

## Conclusions

Our research was conducted on black locust trees that were several decades old. They were particularly valuable because of their beneficial effect on the urban environment and historical value. The results point to the existence of different factors reinforcing each other's significance and causing a decline in the overall health of the examined urban trees.

The concomitance of biotic and abiotic stress factors causes the species–which is widely recommended for difficult urban conditions, especially in places along streets–to lose its resistance. This is partly caused by climate changes, which affect the expansiveness of pathogens and pests, extend the period of plant colonization by pests and increase their numbers of generations. These adverse effects may exert even greater influence on new trees planted in cities and on species that are considered more sensitive than putatively resistant black locust.
